# Circulating and Endometrial Tissue microRNA Markers Associated with Endometrial Cancer Diagnosis, Prognosis, and Response to Treatment

**DOI:** 10.3390/cancers15102686

**Published:** 2023-05-10

**Authors:** Sergio Antonio Oropeza-de Lara, Idalia Garza-Veloz, Bertha Berthaud-González, Margarita L. Martinez-Fierro

**Affiliations:** 1 Molecular Medicine Laboratory, Academic Unit of Human Medicine and Health Sciences, Universidad Autónoma de Zacatecas, Carretera Zacatecas-Guadalajara Km 6, Ejido La Escondida, Zacatecas 98160, Mexico; sergioantoniooropeza@gmail.com (S.A.O.-d.L.); idaliagv@uaz.edu.mx (I.G.-V.); 2 Hospital General Zacatecas “Luz González Cosío”, Servicios de Salud de Zacatecas, Zacatecas 98160, Mexico; bberthaud@hotmail.com

**Keywords:** endometrial cancer, miRNAs, biomarkers, diagnosis, prognosis, response to treatment

## Abstract

**Simple Summary:**

Endometrial cancer (EC) is one of the most common gynecological malignancies. Therefore, it is of great clinical importance to identify potential candidates for diagnostic and prognostic biomarkers in order to identify high-risk patients and obtain a more accurate prognosis in a timely manner. MicroRNAs (miRs) are small single-stranded RNAs that regulate gene expression and play a role in all steps of cancer development. The miRs expressed in endometrial tumor tissue are probably involved in cell proliferation and differentiation, apoptosis, and carcinogenesis. We reviewed the literature to identify potential miRs that may function as diagnostic, prognostic, or response to treatment markers in EC.

**Abstract:**

In developed countries, endometrial cancer (EC) is one of the most common neoplasms of the female reproductive system. MicroRNAs (miRs) are a class of single-stranded noncoding RNA molecules with lengths of 19–25 nucleotides that bind to target messenger RNA (mRNA) to regulate post-transcriptional gene expression. Although there is a large amount of research focused on identifying miRs with a diagnostic, prognostic, or response to treatment capacity in EC, these studies differ in terms of experimental methodology, types of samples used, selection criteria, and results obtained. Hence, there is a large amount of heterogeneous information that makes it difficult to identify potential miR biomarkers. We aimed to summarize the current knowledge on miRs that have been shown to be the most suitable potential markers for EC. We searched PubMed and Google Scholar without date restrictions or filters. We described 138 miRs with potential diagnostic, prognostic, or treatment response potential in EC. Seven diagnostic panels showed higher sensitivity and specificity for the diagnosis of EC than individual miRs. We further identified miRs up- or downregulated depending on the FIGO stage, precursor lesions, and staging after surgery, which provides insight into which miRs are expressed chronologically depending on the disease stage and/or that are modulated depending on the tumor grade based on histopathological evaluation.

## 1. Introduction

In developed countries, endometrial cancer (EC) is one of the most frequent tumors of the female reproductive tract. Worldwide, EC was diagnosed in 417,367 women in 2020, with the highest disease burden in North America and Western Europe. In the same year, EC was the fourth most common female neoplasm in Europe, with an incidence of 12.9–20.2 and a mortality of 2.0–3.7 cases per 100,000 women [1]. According to data obtained from Cancer statistics, 2022, 65,950 new cases of EC were estimated, representing 3.5% of all new cancer cases in the USA, and the disease was fatal for 12,550 patients, which was equivalent to 2.1% of all deaths because of cancer [2].

EC is a heterogeneous malignant disease comprising several histological types, which differ from each other in terms of pathophysiology, clinical manifestations, and prognosis; this heterogeneity hinders the development of screening and treatment strategies. Histomorphological examinations are essential in the diagnosis of EC, but the classification of EC by histomorphological criteria has limited reproducibility, especially in high-grade tumors [3]. EC is currently classified according to The Cancer Genome Atlas (TCGA) criteria and post-TCGA publications. The TCGA applied methods that were too costly and confusing to be systematically applied in clinical medical practice [4]. Since TCGA, two research teams have developed more pragmatic and useful approaches that allow more accessible molecular subtyping in pathology laboratories [5,6]. The European Society of Gynaecological Oncology (ESGO), the European Society for Radiotherapy and Oncology (ESTRO), and the European Society of Pathology (ESP) guidelines classified EC into (i) ultra-mutated ECs characterized by pathogenic variants in the exonuclease domain of DNA polymerase-epsilon (POLE), (ii) microsatellite instability (MSI) hypermutated/mismatch repair (MMR) deficient, (iii) a copy-number high group with frequent TP53 mutations (p53 mutant immunoreactive pattern), (iv) low copy number/non-specific molecular profile (retained MMR protein immunoreactivity and p53 wild-type immunoreactive pattern), and (v) multiple classifiers (combination of markers included in the previous categories) [7,8]. Molecular classification can be performed using the diagnostic algorithm provided by Vermij et al. [9]; the diagnostic requires testing of three immunohistochemical markers (p53, MSH-6, PMS-2) and somatic mutation analysis of POLE (exons 9, 11, 13, 14). Table 1 shows the main characteristics between different subtypes of tumors, from molecular and histological alterations to associated clinical features, including diagnosis and prognosis [4,5,6].

A biopsy of the endometrial lining for pathological examination is the definitive test to confirm an EC diagnosis. The WHO classification (based on the cytomorphological characteristics) distinguishes between endometrial hyperplasia without atypia (EH) and atypical endometrial hyperplasia/endometrioid intraepithelial neoplasia (AEH/EIN). AEH/EIN is characterized by crowded glands with cytologically atypical epithelium separated by little intervening stroma. Cellular atypia is characterized by nuclear enlargement and rounding, pleomorphism, loss of polarity, and the presence of nucleoli [10]. The specific treatment depends on the stage, according to the International Federation of Gynecology and Obstetrics (FIGO), but in general, a hysterectomy with bilateral salpingo-oophorectomy is performed, with dissection of the pelvic and para-aortic nodes, which may be followed by adjuvant chemotherapy (cisplatin-based) with or without radiotherapy [11]. When endometrioid endometrial carcinoma (EEC) is diagnosed at an early stage, surgery typically results in a good prognosis. However, patients with advanced stages of EEC exhibit more aggressive characteristics and an increased risk of metastasis (a multi-step process by which cancer cells from the primary tumor proliferate to form distant secondary tumors) [12,13]. Although important advances have been made in therapeutic strategies, the management of cases with advanced stages of the disease remains difficult, as the 5-year survival is only 10–29%. Thus, there is a need to identify and implement biomarkers that allow early and timely detection to prevent the progression of the disease to advanced stages and to objectively distinguish between histological subtypes. This approach should facilitate research and treatment of specific subtypes of EC [14,15].

MicroRNAs (miRs), a class of short noncoding RNAs with a length of 19–25 nucleotides, regulate gene expression via antisense complementarity or complementarity with specific messenger RNA (mRNA) [16]. Under normal physiological conditions, individual miRs show strict tissue-specific and development stage-specific expression patterns. In contrast, miRs display unique expression profiles, depending on clinical features, in several cancers, including breast cancer, lung cancer, and chronic lymphocytic leukemia [17,18].

MiRs are associated with the development of tumors and may be classified as oncogenic miRs (oncomiRs) and tumor-suppressor miRs. An oncomiR acts as an oncogene and has increased expression in tumor cells, while a tumor suppressor miR acts as a tumor suppressor gene and has decreased expression in tumor cells [19]. The miRs expressed in endometrial tumor tissue are probably involved in cell proliferation and differentiation, apoptosis, and carcinogenesis [20,21]. Our main objective in this narrative review is to identify potential miRs that may serve as diagnostic, response to treatment, and/or prognostic biomarkers of EC in relation to the clinical and histopathological features of the disease.

## 2. Methods

To identify the most suitable miRs that may serve as biomarkers in EC in relation to the clinical and histopathological features of the disease, we searched PubMed and Google Scholar without date restrictions or filters using the following keywords: circulating miRNAs, endometrial tissue miRNAs, endometrial cancer, biomarkers, diagnosis, response to treatment, and prognosis. We included two types of articles: (1) Original articles that were published in English and whose objective was to investigate miRs associated with EC in samples of endometrial tumor tissue, serum, plasma, cell lines, and in silico, in addition to providing statistically significant results (*p* < 0.05). (2) Review articles and guidelines to supplement general information on EC, in particular, epidemiological information and definitions of concepts. We excluded articles that were not in English, miRs obtained from other biological samples, or studies that did not obtain statistically significant results (*p* > 0.05). We classified the miRs according to their potential use in EC in the following categories: diagnostic markers, prognostic markers, and response to treatment. We included 138 original studies, the majority of which are retrospective studies with a sample size of <160 patients. We also included 12 reviews, the World Health Organization (WHO) classification of female genital tumors and the ESGO/ESTRO/ESP guidelines for the management of patients with endometrial carcinoma, which described the expression levels of 138 miRs in EC.

## 3. Results

### 3.1. Diagnostic and Classificatory Capacity of miRs for EC

Numerous promising circulating miRs (found in serum, plasma, and venous blood) have been identified that could contribute to the clinical or histopathological diagnosis of EC. Depending on the type of study, different quantitative methods are used to determine the expression level of each miR and to evaluate their relationship to the disease [22]. In the same way, the tumor tissue miRs profile has been used to evaluate the classificatory capacity of the tumor stage, grade, and progression.

In the following subsections, we group the potential miRs associated with EC according to the matrix in which they have been detected, as well as their diagnostic and/or classificatory capacity. We also discuss their clinical significance.

#### 3.1.1. miRs Studied in Plasma

Blood plasma serves as the liquid base for whole blood and contains 91% to 92% of water and 8% to 9% of solids (coagulants, proteins, electrolytes, vitamins, hormones, nucleic acids, etc.). In adults, liver cells, the bone marrow, degenerating blood cells, general body tissue cells, and the spleen contribute to the formation of plasma content. Plasma content is frequently used to identify markers in a large number of human diseases. In EC and with the aim of identifying miR biomarkers in plasma to assist the clinical screening of disease, Fan et al. [22] conducted a four-phase study in which they analyzed a total of 93 plasma samples from patients with EC who were diagnosed via histopathological examination and who did not accept a therapeutic intervention such as surgery, radiotherapy, or drug treatment prior to sampling. The control group (CG) comprised 79 samples from patients who were randomly recruited and guaranteed to be free of EC after imaging examination. They reported the expression level of three miRs based on quantitative real-time polymerase chain reaction (qRT-PCR). miR-142-3p (*p* = 0.0003), miR-146a-5p (*p* = 0.0002), and miR-151a-5p (*p* = 0.0018) were significantly overexpressed in the plasma of patients with the EC compared with the CG. However, a combination of miRs could provide stronger differentiation power than an individual miR. The three miRs were combined as a panel, and the area under the receiver operating characteristic (ROC) curve (AUC) was 0.716 (95% confidence interval [CI]: 0.640–0.793, sensitivity = 62%, specificity = 64.5%, cutoff = 0.528). Based on the analysis of FIGO stages, there was no significant difference in the expression of the three miRs in the early stage (I: 90% of patients) and advanced stages (II + III + IV: 10% of patients) of EC. The expression of miR-142-3p and miR-146a-5p in pre-menopausal patients with EC was significantly higher than in post-menopausal patients. Compared with controls, miR-142-3p and miR-146a-5p have also been reported as overrepresented in the plasma of patients with EC at diagnosis in the other two studies [22,23,24].

#### 3.1.2. Serum miRs

In a study including 194 patients—92 with a pathological diagnosis of EC (who had not received any type of therapy) and 102 control patients (the CG, without EC or any other type of systemic disease)—the authors found five miRs that were significantly overexpressed in the serum of patients with EC compared with the CG: miR-195-5p, miR-20b-5p, miR-204-5p, miR-423-3p, and miR-484 (fold change [FC] = 1.59–6.21, *p* < 0.0005). Meanwhile, miR-143-3p was downregulated (*p* < 0.0001, FC = −10.11) [25]. After constructing ROC curves using the six-miR panel for patients with early (I) and late (II + III + IV) FIGO stages, the AUC was 0.769 and 0.844, respectively, compared with the CG. The six-miR panel could distinguish patients with early- or late-stage EC from the CG, with a sensitivity of 60.2% and a specificity of 84.3% in early-stage patients and a sensitivity of 66.7% and a specificity of 92.2% in late-stage patients. Another pathological characteristic studied was tumor grade. The AUC of the six-miR panel was 0.773 (sensitivity = 62.9%, specificity = 81.4%) for grade I, 0.766 (sensitivity = 69.8%, specificity = 70.6%) for grade II, and 0.874 (sensitivity = 91.7%, specificity = 71.6%) for grade III. These values demonstrate the ability of this panel to distinguish patients with EC at any stage from the CG. However, there were no significant differences between the different subgroups of patients with EC, indicating that the expression of miRs does not change according to changes in patient characteristics. Compared with previously identified biomarkers, the six-miR panel showed superior performance in diagnosing EC [25]. At diagnosis, serum miRs with more consistent differences between groups of patients with EC and controls include miR-195-5p [25,26,27], miR-20b-5p [28,29,30,31,32,33,34,35,36,37], and miR-204 [38,39]. The serum concentration of these miRs has been reported as increased in patients with EC.

#### 3.1.3. miRs Studied in Endometrial Tissue

The human endometrium consists of glandular epithelium and vascularised stroma and is unique because it is constantly shed and regrown with each menstrual cycle, generating up to 10 mm of new mucosa. Consequently, there are marked changes in cell composition and gene expression across the menstrual cycle, and the expression of many genes is influenced by genetic variation among individuals. Hence, the identification of a consensus of EC tissue biomarkers is quite complex. In addition, the sample sizes for studies that have examined the endometrium and/or EC are modest, and there are important differences between study designs, preservation of the tissue sample, and the staging of the tumor to be evaluated. However, the growing evidence of the miRs involved in carcinogenesis has allowed researchers to propose panels with potential value for diagnosis, prognosis, staging, and treatment response in EC.

##### Tissue miRs with Diagnostic Usefulness for EC

With the aim to identify a set of EEC-associated miRs in tissue and to evaluate their clinical significance, Tsukamoto et al. [20] identified, by next-generation sequencing (NGS), 11 candidate EEC-associated miRs (≥2 times up- or downregulated compared with normal endometrium [NE] tissue, which came from patients who underwent hysterectomy due to uterine myomas). They then evaluated expression levels of those miRs in 28 EEC and 14 NE samples by qRT-PCR. Eight miRs showed significantly different expressions and were EEC-associated miRs in tissue. Three were upregulated—miR-499, miR-135b, and miR-205 (*p* = 0.003, *p* < 0.001, and *p* = 0.002, respectively)—and five were downregulated—miR-10b, miR-195, miR-30a-5p, miR-30a-3p, and miR-21 (*p* = 0.006, *p* < 0.001, *p* = 0.019, *p* = 0.001, and *p* = 0.011, respectively). ROC analysis revealed that two panels of miRs (miR135b/miR195 and miR135b/miR30a-3p) provided a higher AUC when compared with individual miRs. The two panels (miR135b/miR195 and miR135b/miR30a-3p) could distinguish between EEC and NE with an AUC of 0.9835 (95% CI: 0.9677–1.0, *p* < 0.048) and 0.9898 (95% CI: 0.9677–1.0, *p* < 0.038), respectively. Lee et al. [40] obtained similar results. They performed qRT-PCR using 75 formalin-fixed, paraffin-embedded (FFPE) tissue blocks of NE (*n* = 10), endometrial hyperplasia (*n* = 43), and EC (*n* = 22) and identified a panel of six miRs (miR-21, miR-182, miR-183, miR-200a, miR-200c, and miR-205) as the best marker to differentiate EC from the other tissues. The panel had an AUC of 0.961, 91% sensitivity, and 94% specificity (*p* < 0.05), while the individual miRs exhibited an AUC of 0.665–0.904, 64–77% sensitivity, and 66–91% specificity (*p* < 0.05). A smaller panel of four miRs (miR-182, miR-183, miR-200a, and miR-200c) was able to identify the EC of AEH with an AUC of 0.957, 95% sensitivity, and 91% specificity. In the context of differentiating malignant lesions from precursor lesions, these findings could set a precedent for future complementary tools that are useful for determining malignancy in endometrial tissue samples.

In another experiment using qRT-PCR in RNA extracted from fresh-frozen endometrial tissue (37 EC, 20 NE resected from postmenopausal patients with benign gynecologic disease, and 4 AEH samples), Boren et al. [41] identified five miRs with decreased in expression in the transition from NE to AEH to EC: miR-let-7i, miR-221, miR-193, miR-152, and miR-30c (*p* < 0.0001). Eight miRs exhibited a relative increase in expression with the transition from NE to AEH to EC: miR-423 (*p* < 0.02); miR-let-7c (*p* < 0.01); and finally, miR-185, miR-106a, miR-181a, miR-103, miR-107, and miR-210 (*p* < 0.0001).

##### Endometrial miRs Expressed According to Specific EC Chronological Stage

**Figure 1:**
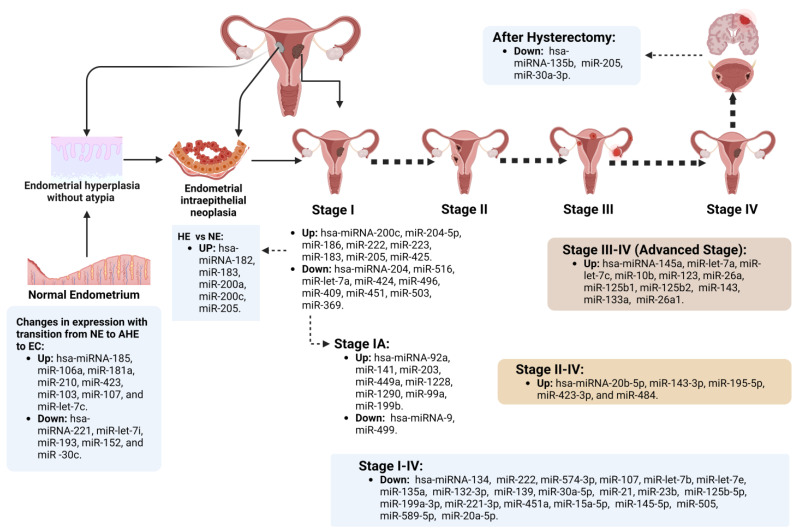
Up- or downregulated microRNAs (miRs) depending on the International Federation of Gynecology and Obstetrics (FIGO) stage, precursor lesions, and after surgical treatment in patients with endometrial cancer (EC). hsa-miR-200a and hsa-miR-200c (members of the miR-200 family) are upregulated in precursor lesions of EC, such as endometrial hyperplasia (EH). In the case of miR-200c, its overexpression is maintained in stage I EC, which could suggest that miR-200 family members act in signaling pathways involved in the carcinogenesis of this tumor [14,20,25,40,41,42,43,44,45,46,47,48,49,50,51,52]. The FIGO stage details are the following: stage I, tumor confined to the uterus; stage II, the tumor has spread from the body of the uterus and grows into the cervical stroma, but it has not spread outside the uterus; stage III, the tumor has spread outside the uterus, but not to the inner lining of the rectum or urinary bladder; and stage IV, the tumor has spread to the rectal mucosa, urinary bladder, or distant organs. AEH: atypical endometrial hyperplasia; EC, endometrial cancer; NE, normal endometrium.

Continuing with the study of the miRs significantly expressed at each chronological stage of the disease, Cohn et al. [42] examined tissue samples from 141 patients with EC. They identified differences in miR expression between EC and NE (20 unmatched endometrial samples), as well as in early EC stages based on qRT-PCR. Several miRs showed >2-fold differences between EC stage I and controls: miR-200c (relative expression ratio [RER] = 3.407), miR-183 (RER = 2.508), miR-205 (RER = 2.202), miR-223 (RER = 2.318), and miR-425 (RER = 2.11), all with *p* < 0.001. In addition, there were differences in the expression of several miRs in stages III–IV compared with stage I (>3 FC and *p* < 0.001), namely miR-145a, miR-let-7a, miR-let-7c, miR-10b, miR-123, miR-26a, miR-125b1, miR-125b2, miR-143, miR-133a, and miR-26a1, with an RER of 3–8.87. Figure 1 shows a summary of the up- and downregulated miRs depending on the FIGO stage in the EC, precursor lesions, and after surgical treatment [14,20,25,40,41,42,43,44,45,46,47,48,49,50,51,52].

##### Tissue miRs Associated with EC, Tumor Grade, and Their Transcriptional Targets

The value of a disease biomarker is its capacity to discriminate between a healthy individual and one with the disease. However, additional value is added if this marker could be useful to understanding the pathogenesis of the disease. Previous in silico studies have proposed miRs related to EC and have proposed target genes and the signaling pathways involved. These miRs include hsa-miR-152, miR-181d, miR-370, miR-495, miR-504, miR-510, miR-543, miR-548e, miR-548v, miR-579, miR-758, and miR-1287, which have been predicted to modulate target genes and signaling pathways associated with EC [21]. However, there are a few studies that have included the identification and validation of EC target genes that may have been used to establish the complete expression profiles for each stage of EC and the modulated signaling pathways [53]. An example is the miR-200 family members. In an effort to evaluate the role of miR-200c in cell growth and drug sensitivity in EC and the underlying mechanisms, Park et al. [54] determined the relative expression of miR-200c in frozen endometrial tissue (24 EC and 7 NE). The authors identified increased expression of this miR in EC (*p* < 0.05). miR-200c regulates the translocation of β-catenin from the cytoplasm to the nucleus via inhibition of bromodomain-containing 7 (BRD7, a validated target of miR-200c), a potential tumor-suppressor gene, resulting in increased expression of its transcriptional target genes, cyclin D1 and c-myc, suggesting its potential role for the EC treatment. Similarly, miR-200a, miR-200b, and miR-429 are highly overexpressed in EEC (FC: 8.29–10.60, *p* < 0.005) and are believed to act as onco-miRs as they have been shown to downregulate phosphatase and tensin homolog (PTEN) in in vitro studies (*p* < 0.05) [55]. PTEN is a negative regulator of the phosphatidylinositol 3-hydroxy kinase/protein kinase B (PI3K/AKT) signaling pathway, which participates in cell growth and survival. It is now well established that PTEN plays a tumor suppressor role in cell proliferation and survival [56]. Mutations in PTEN have been found in 30–80% of EC cases, suggesting that alterations in PTEN occur at a relatively early stage of endometrial tumorigenesis. Hence, characterizing the complex relationship between miRs and the PTEN target gene in endometrial lesions may help to better define some of the molecular pathways driving carcinogenesis [40].

The miR-200 family also exhibits significant expression differences depending on the EC histological type. Dong et al. [57] found that miR-200a was highly overexpressed in the non-EEC subtype compared with EEC (*p* = 0. 025). They also evaluated the expression level of miR-200a/miR-141 and miR-205 by qRT-PCR in 154 FFPE EC tissue samples (102 EEC, 52 serous subtypes [SEC]) and 26 NE samples as controls. They found that miR-200a, miR-141, and miR-205 were significantly increased in EEC (88.2%, 90.2%, and 90.2%, respectively) and non-EEC (86.5%, 75.0%, and 84.6%, respectively) compared with NE (*p* < 0.05) [57]. miRs belonging to the miR-200 family are significantly upregulated in EC, and their modulation is more pronounced in early clinical stages. On the other hand, their expression decreases in more advanced stages and in poorly differentiated tumors [14]. miR-200a and miR-205 are responsible for repressing zinc finger E-box binding homeobox 1 and 2 (ZEB1 and ZEB2), as well as other mesenchymal genes. These miRs are considered custodians of the epithelial phenotype, and loss of these miRs is a marker of aggressiveness and metastasis in EC and other tumors, such as breast and ovarian cancer, based on cellular models [58,59].

In EEC, DNA methyltransferase 3B (DNMT3B) overexpression occurs more often in the subgroups with miR-145 and miR-143 downregulation, and there is a significant correlation between DNMT3B and miR-145 status (*p* = 0.021); this correlation is not significant in non-EEC (*p* ≥ 0.05). This indicates that DNMT3B is a molecular target of these two miRs, which is consistent with in silico analysis because both miRs have complementary sites in the 3′-untranslated region (UTR) and coding region. DNMT3B is necessary for de novo methylation and may be involved in the aberrant methylation in some tumors. By using a database of twenty FFPE EC samples (fifteen low-grade and five high-grade) and five samples as the CG (proliferative endometrium), Zhang et al. [60] assessed by qRT-PCR miR-145 and miR-143 expression. They were downregulated in EC compared with the CG (*p* = 0.034 and *p* = 0.022, respectively). In addition, these miRs were significantly lower in EEC than in non-EEC (*p* ≤ 0.05) [60,61,62].

Another study evaluated tissue samples from 82 patients: 62 had a diagnosis of EC and 20 were controls (non-neoplastic endometrium). The authors focused on discriminating miR profiles between well-differentiated and poorly differentiated EEC and between EEC and SEC. They found decreased expression of miR-125b-5p, miR-let-7c-5p, miR-23b-3p, and miR-99a-5p in grade 3 compared with grade 1 EEC (*p* < 0.05, FC 0.4037–0.4866). They also found significantly downregulated miR-195-5p, miR-34a-5p, miR-let-7g-5p, and miR-497-5p expression in SEC. The above-mentioned miRs target genes involved in the PI3K/AKT and mitogen-activated protein kinase (MAPK) signaling pathway, respectively [63].

**Figure 2:**
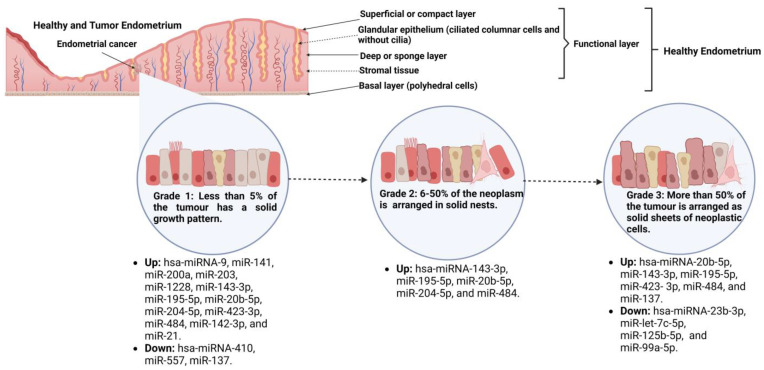
Up- or downregulation of microRNAs (miRs) depending on the endometrial cancer tumor grade. miR-200a, part of the miR-200 family, is one of the miRs mainly overexpressed in grade 1 EC, whereas miR-143-3p remains overexpressed in all three tumor grades [14,20,22,25,63].

Figure 2 summarizes the miRs that are up- or downregulated depending on the EC tumor grade [14,20,22,25,63].

##### Tissue miRs Associated with the EC Histological Types

**Figure 3:**
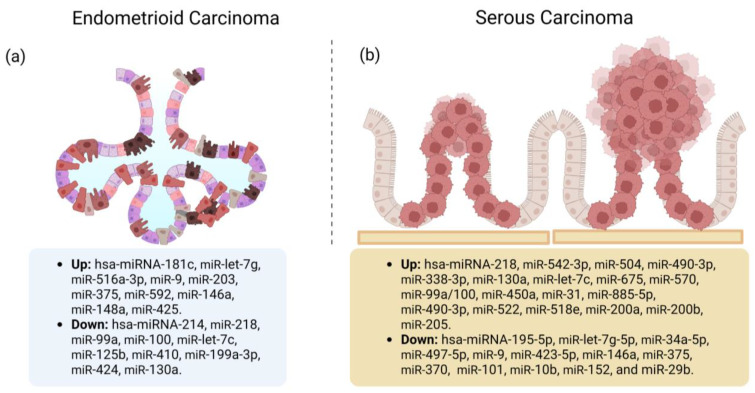
Up- or downregulated microRNAs (miRs) in (**a**) endometrioid endometrial carcinoma (EEC) or (**b**) serous endometrial carcinoma (SEC). Some miRs may be up- or downregulated depending on the histological type. For example, miR-9 is overexpressed in EEC but downregulated in SEC. This could indicate that miR plays different roles depending on the histological type [63,64,66].

Devor et al. [64] evaluated frozen endometrial tissue and identified miRs able to distinguish SEC from EEC. They found that miR-9, miR-423-5p, miR-146a, and miR-375 (FC −232.7 to −4.6, *p* < 0.05) were downregulated and miR-218, miR-542-3p, miR-490-3p, miR-504, miR-338-3p, miR-130a, miR-let-7c, miR-675, miR-570, and miR-518e were upregulated (FC 3.2–80.2, *p* < 0.05). Hence, EEC and SEC have profiles containing shared, unique, and differentiating miRs. EEC is characterized by a histological structure that displays a glandular or villoglandular architecture lined by stratified columnar epithelium with a crowded, complex, and branching architecture. The lining cells are usually columnar and share a common apical border with adjacent cells, resulting in a smoothly contoured glandular lumen. SEC, on the other hand, shows a complex papillary architecture. The papillae vary from short, branched, and hyalinized to long, thin, and delicate [10]. miR-497-5p was also downregulated in EEC (FC = 0.256, 95% CI: 0.180–0.351, *p* < 0.001) and SEC (FC = 0.081, 95% CI: 0.054–0.115, *p* < 0.001) compared with non-tumor tissue (the CG). In addition, differential expression of miR-497-5p distinguished SEC from EEC (FC = 0.318, 95% CI: 0.204–0.480, *p* < 0.001), and there was lower miR-497-5p expression in the hormone receptor-negative and TP53-positive with high Ki-67 expression groups [65]. Figure 3 summarizes the studies reporting up- or downregulated miRs in EEC or SEC [63,64,66].

### 3.2. miRs and EC Prognosis

Prognosis means predicting a likely future course of events—in this case, estimating the future course of a patient’s disease. A prognosis is based on statistics and probability, indices, survival curves, and prognostic scores, with the intent of being an objective and neutral description of the reality of a disease [67].

The surgical stage is the most important prognostic factor, and the information derived from the surgical stage is categorized according to the 2009 FIGO classification [68]. Other prognostic factors in EC are histological type and grade, age, and tumor size. Favorable prognostic factors include FIGO early stage, low-grade, endometrioid, diploid, and hormone receptor-positive EC. Unfavorable prognostic factors include FIGO advanced stage, non-endometrioid, high-grade, and aneuploid EC [12,68,69]. The molecular classification adds another layer of information to the conventional morphologic features, and considering the high number of possible markers, only a few have been included in internationally recommended guidelines for risk stratification. In 2020, the ESGO–ESTRO–ESP guidelines encouraged the determination of the molecular subtype and classification of all EC patients. Table 2 includes the prognostic risk groups to guide adjuvant therapy use according to the ESGO–ESTRO–ESP guidelines [8]. Approximately 75% of patients with EC are diagnosed at FIGO stage I or II, and the 5-year overall survival ranges from 74% to 91%; for FIGO stages III and IV, the 5-year overall survival is 60% and 20%, respectively. Regarding lymph node metastasis, the 5-year disease-free survival rate is estimated at 90% for patients without metastasis, 60–70% if there is pelvic lymph node metastasis, and 30–40% if there is paraaortic lymph node metastasis [68,70].

In EC, miR-205 upregulation is associated with poor survival and promotes cell proliferation and invasion by targeting PTEN and ESRRG, respectively, suggesting that it may function as a marker of poor prognosis [71,72]. On the other side, Wilczynski et al. [73] found that miR-205 expression levels were higher in tumors with less than half myometrial invasion and non-advanced EC (*p* = 0.039, *p* = 0.045, respectively). Kaplan–Meier analysis revealed that higher levels of miR-205 were associated with better overall survival (hazard ratio [HR] = 0.34, 95% CI: 0.21–0.82, log-rank test *p* = 0.034). Hence, miR-205 works as a marker of good prognosis. In addition, miR-205 was most commonly upregulated in non-EEC without lymph node metastasis (*p* = 0.030), but such association was not present in EEC. Additional studies are needed to elucidate the signaling pathways through which miR-205 acts to characterize its prognostic utility. There are contradictory results because while some authors find that miR-205 is a possible marker of poor prognosis, others find that it may function as a marker of good prognosis. Dong et al. [57] found that miR-205 expression levels were higher in the estrogen and progesterone receptor (ER/PR)-positive subgroups, and the association between miR-205 and PR reached statistical significance (*p* = 0.024). ER/PR positivity is considered a good prognostic factor because these tumors are considered to be hormone-sensitive, which is a characteristic of type I endometrial tumors [69].

SEC is considered an aggressive tumor with a high relapse rate, early and deep myometrial invasion, and frequent lymph vascular space involvement [74,75]. Based on Kaplan–Meier analysis, researchers have found that miR-101, miR-10b, miR-139-5p, miR-152, miR-29b, and miR-455-5p downregulation correlated with decreased overall survival (*p* < 0.05); miR-152, miR-29b, and miR-455-5p downregulation correlated with decreased progression-free survival (*p* < 0.05); and miR-10b, miR-29b, and miR-455-5p downregulation correlated with vascular invasion (*p* = 0.048, *p* = 0.013, and *p* = 0.032, respectively) [66]. These findings suggest that their downregulation occurs during the course of tumor progression and, particularly, during the acquisition of cancer metastatic potential. This information could be useful to help predict a patient’s risk for vascular invasion when conclusive results are not available.

Insulin-like growth factor 1 receptor (IGF1R) is a target gene of miR-625-5p (determined based on the transfection of the miR-625-5p inhibitor in HEC-1-B cells). Therefore, it could be involved in regulating cell proliferation and migration in EC by activating the PI3K/Akt signaling pathway. Patients with low expression of miR-625-5p showed significantly better overall survival compared with those with high miR-625-5p expression (Kaplan–Meier analysis, *p* = 0.0287), suggesting that miR-625-5p acts as an onco-miR and accelerates EC progression by activating the IGF1R/PI3K/Akt pathway [76]. IGF1R is a transmembrane tyrosine kinase receptor implicated in intracellular signaling pathways, such as PI3K/Akt [77].

miR-497-5p participates in EC progression and could regulate the phosphatidylinositol 4-kinase beta (PI4KB)/hedgehog signaling pathway. In EC, miR-497-5p mimics decreased the expression of PI4KB and hedgehog in HEC-1B cells (*p* < 0.01), while miR-497-5p inhibitors increased PI4KB and hedgehog expression in HEC-1A cells (*p* < 0.01) [78]. miR-497-5p transcriptional targets have been identified in other tumor types. For example, Raf-1 serine/threonine kinase (RAF1), kinase insert domain receptor/vascular endothelial growth factor receptor 2 (KDR/VEGFR-2), and IGF1R—which represent genes involved in the MAPK pathway (regulates cell growth and proliferation)—have been identified in renal, non-small-cell lung, and hepatocellular cancer [79,80,81]. It seems that the miR-497-5p expression level correlates with disease severity. In one study, the authors reported that decreased miR-497-5p expression and increased expression of the target gene empty spiracles homeobox 1 (EMX1) were significantly associated with advanced clinical and histopathological characteristics (stage, grade, and histology) of EC (*p* < 0.05). In addition, there was a worse prognosis and poor overall survival (HR = 0.536, 95% CI: 0.345–0.831, *p* = 0.005) based on Kaplan–Meier survival analysis [82]. The decreased miR-497-5p expression has been observed in women with EC recurrence compared with women without recurrence (FC = −3.04, FC cut-off = 0.45, *p* = 0.019). Patients with a miR-497-5p FC < 0.45 were more likely to show recurrence (*n* = 4; 80%) compared with those with an FC > 0.45 (*n* = 3; 19%), *p* = 0.025 [83]. Liu et al. [84] showed that miR-497 and miR-16-5p were downregulated while the mechanistic target of rapamycin kinase (mTOR) was upregulated in EC tissue compared with healthy adjacent tissue (*p* < 0.05). The authors suggested that hsa_circ_0011324 overexpression affects the expression of miR-497-5p and miR-16-5p and therefore promotes proliferation, migration, and invasion.

miR-298 acts as a tumor-suppressor miR. In one study, miR-298 overexpression suppressed Ishikawa cell proliferation and invasion, but these changes were abolished by catenin delta 1 (CTNND1) overexpression (*p* < 0.05). Moreover, miR-298 overexpression reduced CTNND1 mRNA and protein levels in Ishikawa cells (*p* < 0.05). These observations suggest that CTNND1 is a direct target of miR-298 in EC progression [85]. CTNND1 is a member of the cadherin–catenin complex and a key regulator of cell–cell adhesion, as it regulates the cell adhesion properties of C-, E-, and N-cadherins. Thus, CTNND1 may function as an oncogene in various tumor types (including EC) by regulating several signaling pathways, such as the Wnt pathway [86].

miR-199a inhibits EC cell metastasis and invasion by targeting family with sequence similarity 83, member B (FAM83B) in the epithelial–mesenchymal transition (EMT) signaling pathway. Hence, overexpression of this miR could be associated with a better prognosis [87]. Patients with stage III–IV EC with miR-199a overexpression had longer median progression-free survival (13.3 versus 26.7 months, *p* < 0.048) and median overall survival (20 versus 40 months, *p* < 0.0068), even though the expression was not significantly different from controls [42].

Table 3 lists the miRs with potential diagnostic, prognostic, or treatment response value for EC (in which its expression was reported with *p* < 0.05), their physiological function, and the associated target genes.

### 3.3. Treatment Response

The principal method of treatment in early-stage EC is surgery (laparotomy or laparoscopy); the extent of surgery includes total hysterectomy with bilateral salpingo-oophorectomy [69]. Sentinel node biopsy is an alternative to lymph node dissection for lymph node staging, and a negative sentinel node is accepted to confirm pN0. Sentinel lymph has a high sensitivity of node status for lymph node staging in patients with early-stage EC (it can be omitted in cases without myometrial invasion) and is associated with a substantially lower risk of post-operative morbidity, especially lower leg lymphedema. Staging infracolic omentectomy should be performed in stage I SEC, carcinosarcoma, and undifferentiated carcinoma, but it can be omitted in clear cell and EEC in stage I disease. For women with AEH/EIN or grade 1 EEC without myometrial invasion and without genetic risk factors who wish to preserve fertility, medroxyprogesterone acetate (400–600 mg/day) is the recommended treatment.

Adjuvant treatment recommendations for EC strongly depend on the prognostic risk group: for patients with low-risk EC, no adjuvant treatment is recommended; for patients with EC stage I–II, low-risk based on POLE-mutation, omission of adjuvant treatment should be considered. In case of intermediate risk, adjuvant brachytherapy decreases vaginal recurrence, and for p53 abnormal carcinomas restricted to a polyp or without myometrial invasion, adjuvant therapy is generally not recommended. Patients in the high–intermediate risk group (pN0 after lymph node staging) can receive adjuvant brachytherapy to decrease vaginal recurrence; in case of substantial lymphovascular space invasion (LVSI) or stage II, external beam radiation therapy (EBRT) can be considered; and if there are patients with substantial LVSI and/or high-grade, adjuvant chemotherapy should be considered. Patients with high–intermediate risk cN0/pNx (lymph node staging not performed) are recommended adjuvant EBRT, especially for substantial LVSI and/or for stage II, additional adjuvant chemotherapy can be considered. Patients with high risk are recommended EBRT with concurrent and adjuvant chemotherapy or alternatively sequential chemotherapy and radiotherapy. In case of advanced disease, maximal cytoreduction should be considered only if macroscopic complete resection is feasible with acceptable morbidity; for unresectable tumors, consider definitive radiotherapy with EBRT and intrauterine brachytherapy or neoadjuvant chemotherapy prior to surgical resection or radiotherapy, depending on response. The combination of carboplatin and paclitaxel is the standard chemotherapy treatment of advanced/recurrent EC, there is no standard of care for second-line chemotherapy, but doxorubicin and paclitaxel are considered the most active therapies [8].

These drugs have different modes of action. The action of cisplatin is associated with its ability to form inter- and intra-strand DNA cross-links causing G1 arrest [120]. Li et al. [121] suggest that the damaging effects of cisplatin on DNA are also associated with the expression of genes involved in apoptosis. Doxorubicin intercalates into DNA, thereby inhibiting macromolecular biosynthesis and generating free radicals and hydrogen peroxide, which activate mitochondria-induced apoptosis [122]. Finally, paclitaxel interferes with normal microtubule growth [123].

#### 3.3.1. Mechanisms of Chemoresistance in EC

The underlying causes of drug resistance in malignant neoplasms are multifactorial. The factors that may influence the favorable or unfavorable response to drugs used in EC are found at the molecular level. Indeed, myriad researchers have observed that the expression or under-expression of certain elements belonging to numerous signaling pathways is related to intrinsic/acquired chemoresistance or chemosensitivity, which in many cases leads to cancer recurrence, resulting in treatment failure and death. Early identification of conditions influencing chemoresistance helps to predict the sensitivity of cancer cells to the drug, optimize therapy, and reduce toxic side effects [124].

Different proteins involved in apoptosis related to chemoresistance have been identified. For example, overexpression of the anti-apoptotic members of the Bcl-2 family, such as Bcl-2 and BCL2-like 1 (Bcl-XL) (intrinsic apoptotic pathway), have been linked to cancer chemoresistance, while elevated levels of pro-apoptotic proteins, such as BCL2 associated X (Bax), promote apoptosis and sensitize tumor cells to various cancer treatments [125,126]. Because TP53 is involved in the control of cell cycle progression, it has been considered an important determinant of sensitivity to chemotherapeutic drugs. Accumulated evidence indicates that the level of TP53 expression could influence paclitaxel sensitivity as a result of increased susceptibility to apoptosis following persistent G2/M arrest [127]. TP53 inactivation is associated with upregulation of the mitochondrial protein Bcl-2 and downregulation of Bax [128]; hence, paclitaxel treatment may modulate the relative levels of these apoptotic regulators (e.g., Bcl-2 phosphorylation) and thereby influence drug resistance and sensitivity through these two proteins [129]. In turn, consistent with previous findings, Bcl-2 upregulation has also been shown to be responsible for acquired chemoresistance to platinum compounds in gynecologic cancers [130].

AKT is a central protein in many cellular pathways, such as cell survival, proliferation, glucose uptake, metabolism, angiogenesis, as well as radiation and drug response [131]. The AKT1 and AKT2 isoforms have been shown to be responsible for the acquisition of resistance against cisplatin and paclitaxel, while all three AKT isoforms increase resistance to doxorubicin in EC cells [132]. It is possible that chemotherapy response rates in EC do not exceed 50% because downregulation or deletion of PTEN (frequently mutated in EC) leads to increased resistance against platinum compounds [133,134,135]. An additional mechanism of resistance to taxanes (including paclitaxel) is the selective overexpression of β-tubulin subtypes such as β-tubulin III and β-V [136], as the presence of β-tubulin III subunits inhibits taxane-promoted subunit assembly. This mechanism may be particularly important because it may predict response to taxanes [137]. Umezu et al. [138] showed by immunohistochemical staining for β-tubulin III that there was increased expression of this protein in histological types of ovarian cancer (clear cell and mucinous) commonly associated with worse response to chemotherapy. Furthermore, tumors with high levels of β-tubulin III did not respond to standard chemotherapy (taxanes).

#### 3.3.2. miRs Involved with Treatment Response in EC

As mentioned previously, surgery is the initial treatment in the early stages of EC. Plasma concentrations of miR-135b, miR-205, and miR-30a-3p decrease significantly after hysterectomy (*p* = 0.003), suggesting that these miRs in plasma are mainly from EEC and NE, and they may serve as a non-invasive biomarker for early detection of relapses, which would indicate failure of surgical treatment [20].

All miR-200 family members are significantly upregulated in EC and most pronounced in early clinical stages, and a systematic decrease in their expression has been noted in higher stages and in poorly differentiated tumors [14]. In an in vitro study, the authors transfected Hec-1A and Ishikawa cells with anti-miR-200c and then treated them with cisplatin (0, 3, and 6 µM for Hec-1A cells and at 1, 2, and 3 µM for Ishikawa cells) for 2 days. They observed that anti-miR-200c induced an additive effect on the cisplatin cytotoxicity (*p* < 0.01 and *p* < 0.05 for days 1 and 2, respectively). They also performed the experiment with paclitaxel, but there was no additive effect on cytotoxicity. Thus, it can be inferred that miR-200c may partially regulate the cytotoxicity of chemotherapeutic agents used to treat EC [54]. Another member of the miR-200 family may also be involved in the regulation of drug sensitivity, as transfection with anti-miR-429 enhanced the cytotoxic effect of cisplatin in HEC-1A cells (*p* < 0.001). Based on the accumulated knowledge, specific inhibition of the miR-200 family using anti-miR could represent a new therapeutic strategy for EEC [139].

One possible mechanism by which various members of the miR-200 family (miR-200b, miR-200c, and miR-429) induce cisplatin resistance is by repressing AP-2α expression in HEC-1A cells [140]. AP-2α functions as a tumor suppressor by regulating the transcription of genes involved in apoptosis and cell proliferation. AP-2α regulates the transcriptional activation of E-cadherin [141] and PTEN [142] and the transcriptional repression of Bcl-2 [143]. The presence of the single nucleotide polymorphism (SNP) rs1045385, with an A > C variation, decreased the binding of miR-200b, miR-200c, and miR-429 to the 3′-UTR of AP-2α, which upregulated AP-2α protein expression and increased cisplatin sensitivity in HEC-1A cells (*p* < 0.01) [140]. In contrast to prior data, there was a significant increase in chemosensitivity to paclitaxel but not to cisplatin in Hec50 cells transfected with miR-200c mimic versus controls (scrambled negative control). The chemosensitivity to 25 nmol/L paclitaxel increased by 37% and 45% in the cells treated with miR-200c mimic versus the mock (transfection reagent only) and negative controls, respectively (*p* < 0.05). These findings suggest that restoration of miR-200c enhances chemosensitivity to microtubule-directed agents [144].

miR-29b enhanced the sensitivity of EC cells to cisplatin and increased cisplatin-induced apoptosis by regulating the expression of BAX and Bcl-2. miR-29b upregulation increased BAX expression and decreased Bcl-2 expression in EC cells. Moreover, miR-29b changed PTEN and p-AKT expression by directly binding to the 3′-UTR of PTEN [145]. Mutations in PTEN have been found in 55% of precancerous lesions, up to 80% of EEC, and up to 90% of high-grade tumors [146]. The survival rate of HEC-1-B and Ishikawa cells decreased after miR-29b upregulation when these cells were treated with cisplatin compared with the negative group (*p* < 0.05). At the same time, the opposite phenomenon was observed when miR-29b was downregulated (*p* < 0.05). These findings suggest that miR-29b enhances the sensitivity of EC cells to cisplatin. Furthermore, miR-29b upregulation increased cisplatin-induced apoptosis in HEC-1-B and Ishikawa cells (*p* = 0.021 and *p* = 0.028, respectively) by increasing caspase 3/7 activity and by regulating the expression of BAX and Bcl-2. Based on these findings, miR-29b might be used as a biomarker to predict clinical response to chemotherapy in EC [145].

Another miR, with an opposite effect to miR-29b, is miR-135a because its overexpression decreased the sensitivity of Hec-1-B and Ishikawa cells after cisplatin treatment (*p* < 0.05). In addition, miR-135a upregulation inhibited cisplatin-induced apoptosis by decreasing the caspase3/7 activity in Hec-1-B and Ishikawa cells (*p* = 0.012, *p* = 0.015, respectively). The Caspase-Glo 3/7 assay was used to examine the effect of miR-135a on cisplatin-induced apoptosis of EC cells. Furthermore, cisplatin-induced apoptosis in EC cells was inhibited by miR-135a: It could regulate BAX and Bcl-2 expression, showing common molecular signaling pathways with miR-29b [147].

miR-625 overexpression sensitizes the response to paclitaxel by regulating ZEB2 in HEC-1 cells (*p* < 0.001). Furthermore, inhibiting miR-625 expression or forced ZEB2 overexpression increased paclitaxel resistance in Ishikawa cells (*p* < 0.001) [148]. ZEB2 is a DNA-binding transcription factor that is mainly involved in EMT and plays a pivotal role in drug resistance, survival, tumor recurrence, and metastasis [149]. The above data revealed that downregulated ZEB2 can make EC cells more sensitive to paclitaxel, and perhaps it can sensitize EC that is refractory to paclitaxel treatment and lead to the downregulation of twist family bHLH transcription factor 1 (Twist1), matrix metalloproteinase-2 (MMP2), and vimentin and upregulation of E-cadherin. [148].

In paclitaxel-resistant EC cells, miR-218 was downregulated compared with the cell lines not resistant to paclitaxel: the relative expression levels were ~71.6% and ~66.5% downregulation in RL95-2 and Ishikawa cells, respectively (*p* < 0.05). Moreover, miR-218 overexpression sensitized drug-resistant EC cells to paclitaxel (*p* < 0.05). High mobility group box 1 (HMGB1) is a target gene of miR-218. HMGB1 was upregulated in paclitaxel-resistant EC cells (*p* < 0.05). It mediated autophagy and contributed to chemotherapy resistance in EC in vitro [150].

Paclitaxel sensitivity was enhanced by miR-34b transfection in the Hec-108, Hec-1B, and KLE cell lines. Treatment with miR-34b changed the half maximal inhibitory concentrations (IC_50_) for paclitaxel from 0.9 × 10^−5^ to 0.8 × 10^−5^ in HEC-108 cells, from 0.3 × 10^−5^ to 1.0 × 10^−6^ in HEC-1B cells, and from 0.4 × 10^−5^ to 1.0 × 10^−6^ in KLE cells. In an in vivo xenograft tumor model, 28 days of treatment with miR-34b and paclitaxel markedly reduced tumor growth compared with treatment with negative control miR and paclitaxel (*p* < 0.05). These data suggest that miR-34b enhances paclitaxel sensitivity in EC cells by downregulation of MET proto-oncogene (MET) expression [151]. MET encodes a receptor tyrosine kinase that is widely expressed in epithelial cells. Increased MET expression has been found in a number of human cancers, implicating MET in their pathogenesis [152].

## 4. Conclusions

In this review, we described 138 miRs with potential diagnostic, prognostic, or treatment response potential in EC. Seven diagnostic panels (one in serum, one in plasma, and five in endometrial tissue) showed higher sensitivity and specificity for the diagnosis of EC than individual miRs. We further identified 85 miRs up- or downregulated depending on the FIGO stage, precursor lesions, and staging after surgery, which provides insight into which miRs are expressed chronologically depending on the disease stage. We identified 30 miRs that are modulated depending on the tumor grade based on histopathologic evaluation and 52 miRs differentially expressed between EEC and SEC. These miRs may have diagnostic adjuvant potential when histopathologic evaluation is inconclusive. Several miRs may have potential prognostic value in EC, one of the most studied is miR-497-5p, as it has been found to regulate cell growth and proliferation, and the expression level correlates with disease severity. Finally, eight miRs (hsa-miR-200c, miR-29b, miR-135a, miR-200b, miR-429, miR-625, miR-218, and miR-34b) are associated with increased or decreased chemosensitivity to cisplatin or paclitaxel, which are two of the chemotherapeutics used to treat EC. These miRs may be useful in the future: Perhaps treatment response to these drugs could be predicted depending on their expression. The wealth of EC-related miR data in the literature clearly demonstrates the need to collect information about all implicated miRs because each study may reflect different disease stages, patient subgroups, and experimental methods, among other factors. Studying the roles of miRs in a complex disease such as EC requires the integration of data from several sources, including miR databases, miR target databases, and the biomedical literature.

### Gaps and Opportunity Areas for the Use of miRs as Markers with Clinical Value in EC

It is important to mention that despite the large amount of existing information on miRs and EC, it currently is not feasible to use miRs during the diagnostic approach of EC because the full characterization to use them as a tool in the medical field is not completely established. As an example, there are no well-established expression cut-off points associated with the disease or with its features or progression stages. In the same way, some of the limitations identified during our literature research are that most of the studies have been retrospective and conducted on small and heterogeneous study populations, the experimental methodology and the type and quality of the samples evaluated in each study differ widely, and therefore, it currently is not possible to establish a consensus of its usefulness in the clinical field. However, the current information integrated into this review may be used as a basis to conduct clinical trials using either one panel or individual miRs to determine the best method for miR extraction (according to the biological sample used), the establishment of cut-off expression values with clinical significance (diagnostic, prognostic, or choice of treatment), and the correct methods for the data obtaining and integration with the recent molecular classifications of EC. Likewise, it is highly desirable to integrate the miRs that have demonstrated clinical usefulness to stratify patients according to treatment or adjuvant treatment modalities and thus try to unify the information generated from these miRs with appropriate classifications used by validated medical guidelines.

## Figures and Tables

**Table 1:** Molecular classification of endometrial carcinoma according to The Cancer Genome Atlas (TCGA) criteria and post-TCGA publications. Adapted from [4,5,6].

Characteristics	POLE-Mutant	MSI	p53wt/NSMP	p53 Abnormal (CN High)
Mutational frequency	>100 mutations/Mb	100–10 mutations/Mb	<10 mutations/Mb	<10 mutations/Mb
Somatic CN alterations	Very low	Low	Low	High
Top five recurrent gene mutations (%)	POLE (100%) DMD (100%) CSMD1 (100%) FAT4 (100%) PTEN (94%)	PTEN (88%) PIK3CA (54%) PIK3R1 (42%) RPL22 (37%) ARID1A (37%)	PTEN (77%) PIK3CA (53%) CTNNB1 (52%) ARID1A (42%) PIK3R1 (33%)	TP53 (92%) PIK3CA (47%) FBXW7 (22%) PPP2R1A (22%) PTEN (10%)
Associated histological feature	Endometrioid Grade 3 Broad front invasion TILs Giant tumoral cells	Endometrioid Grade 3 LVSI substantial MELF-type invasion TILs	Endometrioid Grade 1–2 Squamous differentiation ER/PR expression	Serous Grade 3 LVSI High cytonuclear atypia Slit-like spaces
Associated clinical features	Lower BMI Early Stage (IA/IB) Early onset	Higher BMI Lynch Syndrome	Higher BMI	Lower BMI Advanced stage Late onset
Prognosis in early stage (I–II)	Excellent	Intermediate	Excellent/intermediate/poor	Poor
Diagnostic test	NGS (exons 9, 13, 14 or 9–14) Tumor mutation burden	MMR-IHC (MLH1, MSH2, MSH6, PMS2) MSI assay Tumor mutation burden		p53-IHC NGS

POLE: polymerase epsilon, MSI: microsatellite instability, CN: copy-number, NSMP: no specific molecular profile, Mb: megabase, DMD: dystrophin, CSMD1: CUB and Sushi multiple domains 1, FAT4: FAT atypical cadherin 4, PTEN: phosphatase and tensin homolog, TP53: tumor protein p53, PIK3CA: phosphatidylinositol-4,5-bisphosphate 3-kinase catalytic subunit alpha, PIK3R1: phosphoinositide-3-kinase regulatory subunit 1, CTNNB1: catenin beta 1, ARID1A: AT-rich interaction domain 1A, FBXW7: F-box and WD repeat domain containing 7, PPP2R1A: protein phosphatase 2 scaffold subunit A alpha, TILs: tumor-infiltrating lymphocytes, LVSI: lymphovascular space invasion, MELF: microcystic elongated and fragmented type of invasion, ER: estrogen receptor, PR: progesterone receptor, BMI: body mass index, NGS: next-generation sequencing, IHC: immunohistochemistry, MMR: mismatch repair, MLH1: mutL homolog 1, MSH2: mutS homolog 2, MSH6: mutS homolog 6, PMS2: PMS1 homolog 2, mismatch repair system component.

**Table 2:** Prognostic risk group according to the ESGO–ESTRO–ESP guidelines [8].

Risk Group	Molecular Classification Unknown	Molecular Classification Known
Low	Stage IA endometrioid, grade 1–2, LVSI negative or focal	Stage I–II POLEmut endometrial carcinoma, no residual disease. Stage IA MMRd/NSMP endometrioid carcinoma + low grade + LVSI negative or focal.
Intermediate	Stage IB endometrioid, grade 1–2, LVSI negative or focal. Stage IA endometrioid, grade 3, LVSI negative or focal Stage IA non-endometrioid (serous, clear cell, undifferentiated carcinoma, carcinosarcoma, mixed) without myometrial invasion	Stage IB MMRd/NSMP endometrioid carcinoma + low-grade + LVSI negative or focal. Stage IA MMRd/NSMP endometrioid carcinoma + high-grade + LVSI negative or focal. Stage IA p53abn and/or non-endometrioid without myometrial invasion.
High-intermediate	Stage I endometrioid, substantial LSVI, regardless of grade and depth of invasion Stage IB endometrioid, grade 3, regardless of LVSI status Stage II	Stage I MMRd/NSMP endometrioid carcinoma + substantial LVSI regardless of grade and depth of invasion. Stage IB MMRd/NSMP endometrioid carcinoma high-grade regardless of LVSI status. Stage II MMRd/NSMP endometrioid carcinoma
High	Stage III–IVA with no residual disease Stage I–IVA non-endometrioid (serous, clear cell, undifferentiated carcinoma, carcinosarcoma, mixed) with myometrial invasion, and with no residual disease	Stage III–IVA MMRd/NSMP endometrioid carcinoma with no residual disease. Stage I–IVA p53abn endometrial carcinoma with myometrial invasion, with no residual disease. Stage I–IVA NSMP/MMRd serous, undifferentiated carcinoma, carcinosarcoma with myometrial invasion, with no residual disease.
Advanced metastatic	Stage III–IVA with residual disease Stage IVB	Stage III–IVA with residual disease of any molecular type. Stage IVB of any molecular type.

ESGO: European Society of Gynaecological Oncology, ESTRO: European Society for Radiotherapy and Oncology, ESP: European Society of Pathology, LVSI: lymphovascular space invasion, MMRd: mismatch repair deficient, NSMP: non-specific molecular profile, p53abn: p53 abnormal, POLEmut: polymerase-mutated.

**Table 3:** The microRNAs (miRs) with diagnostic, prognostic, or treatment response marker potential in endometrial cancer (EC) (with significant expression differences, *p* < 0.05).

miR	Function in EC	Sample	Expression	Target Genes	Clinical Value	Reference
hsa-miR-143-3p	Tumor-suppressive factor by regulating tumorigenesis and progression.	Serum	Up	MAPK1	Diagnosis	[25]
hsa-miR-143-3p	Might inhibit cell proliferation, metastasis, and promote the apoptosis of EC cells.	ET	Down	MAPK1	Prognosis	[88]
hsa-miR-423	Inhibit cisplatin-induced apoptosis from decreasing the sensitivity of EC.	EC cell lines: HEC-1B and Ishikawa cells	Up	Bcl-2, Caspase 3/7	Treatment response	[89]
hsa-miR-142-3p	Mediation of cell apoptosis by the miR-142-3p-FAM98A signaling pathway (anti-apoptotic and pro-proliferative effects).	Plasma	Up	FAM98A	Diagnosis	[22,23]
hsa-miR-146a-5p	Attenuates the effect of NIFK-AS1 on M2 polarization inhibition of macrophages and estrogen-induced EC cell proliferation, migration, and invasion.	Plasma	Up	NIFK-AS1	Diagnosis	[22,24]
hsa-miR-151a-5p *	Induce proliferation, migration, and partial epithelial metastasis.	Plasma	Up	E-cadherin Fibronectin SNAI2	Diagnosis	[22,90]
hsa-miR-195-5p	Suppressing cell migration, proliferation, and promote apoptosis.	Serum	Up	PI3K/AKT and MAPK/ ERK pathways FGFR1, FGF2	Diagnosis	[25,26,27]
hsa-miR-20b-5p	VEGFA transcription.	Serum	Up	HIF1A PTEN STAT3	Diagnosis	[28,29,30,31,32,33,34,35,36,37]
hsa-miR-204	Mediates the migration and invasion of EC by regulating FOXC1.	- Serum - EC cell lines: HEC1A, HEC1B, AN3CA, KLE, RL95 - ET	Up	*FOXC1*	Diagnosis	[38,39]
hsa-miR-484 *	Tumor suppressor.	MCF7 and T-47D cells	Down	KLF4	Treatment response	[91]
hsa-miR-499	Suppressed tumor growth and angiogenesis.	ET	Up/Down	VAV3	Diagnosis	[20,92]
hsa-miR-135b	Promotes proliferation of EC cells.	ET	Up	FOXO1	Diagnosis	[20,93]
hsa-miR-205	Tumor suppressor through the inhibition of EMT.	ET	Up	PTEN	Prognosis	[94,95]
hsa-miR-10b	Inhibits apoptosis and promotes proliferation, migration, and invasion of EC cells.	ET	Down	HOXB3	Diagnosis	[96]
hsa-miR-195	Inhibits migration, invasion and EMT.	- ET - AN3-CA and Hec1A cells	Down	SOX4 GPER or GPR30	Diagnosis	[97,98]
hsa-miR-30a-5p *	Inhibits cell proliferation and migration.	ET	Down	UBE3C	Diagnosis	[99]
hsa-miR-30a-3p	Modulate autophagy.	ET	Down	BECN1	Diagnosis	[20,100]
hsa-miR-let 7a	Inhibits the growth of EC cells.	- ET - HeLa cells	Down	AURKB	Diagnosis	[101]
hsa-miR-221	Act similar to a critical site of the regulatory pathway ERα/HIF1-α/SNAI2.	ET	Up/Down	LMOD1 ERα MDM2	Diagnosis	[41,102]
hsa-miR-193 *	Enhance cell invasion-mediated EMT and improve cell proliferation through the ING5/PI3K/AKT signal pathway.	ET	Up/Down	RAMP1 ING5	Diagnosis	[41,103]
hsa-miR-152	Inhibits proliferation of EC cells via inducing G2/M phase arrest by suppressing CDC25B expression.	- ET - KLE and HEC-1B cells	Down	ENPP2 SNCAIP CDC25B	Diagnosis	[41,104]
hsa-miR-30c	Modulates MTA1, which may promote EC progression through the AKT/mTOR/4E-BP1 pathway.	- ET - EC cell lines: Ishikawa, HEC-1B, and RL-952	Down	GPRASP2 MTA1	Prognosis	[41,105]
hsa-miR-185 *	Inhibited EMT by targeting Rab25 expression.	ET	Up	KLF2 Rab25	Diagnosis	[41,106]
hsa-miR-106a	Acts as an oncogenic miR in EC by inhibiting tumor suppressor BCL2L11 expression.	ET	Up	TGFB1I1 BCL2L11	Diagnosis	[41,107]
hsa-miR-181a	Acts as an oncogenic miR that negatively regulate tumor suppressor PTEN.	ET	Up	PTEN DPP6	Diagnosis	[41,108]
hsa-miR-210	Promoted the progression of EC by negative regulation NFIX expression.	ET	Up	ENPP2 C2orf32 NFIX	Diagnosis/Prognosis	[41,109]
hsa-miR-103	- Regulates the progression in EC through ZO-1. - Regulates the growth and invasion of EC cells through the downregulation of TIMP-3	- ET - Cell lines: HEC-1B and Ishikawa	Up	ZO-1 TIMP-3	Diagnosis/Prognosis	[110,111]
hsa-miR-let 7c	Contributes to paclitaxel resistance via Aurora-B in SEC.	SEC cell lines: USPC1, USPC1-PTXR, USPC1-PTXR2	Down	Aurora-B	Treatment response	[112]
hsa-miR-200c	- It is speculated that the increase in cell proliferation is mediated through repression of KLF9. - Regulated the translocation of β-catenin from the cytoplasm to the nucleus via inhibition of BRD7, resulting in increased expression of its transcriptional target genes, cyclin D1 and c-myc.	- ET - Cell lines: HEC-1A, Ishikawa	Up	PTEN KLF9 BRD7	Diagnosis	[42,54,113]
hsa-miR-183	Promotes cell proliferation and invasion by targeting MMP-9.	- ET - EC cell lines: KLE, HEC-1-A and HHUA	Up	MMP-9	Diagnosis	[114]
hsa-miR-186	May reduce the expression of tumor suppressor FOXO1 and thereby deregulates cell cycle control.	- Serum - EC cell lines: HEC-1B and Ishikawa	Up	FOXO1	Diagnosis	[43,115]
hsa-miR-141-3p	Promoter EC cells proliferation, indicating that could act as an oncogenic miR in EC progression.	- ET - EC cell lines: HEC-1 A and KLE cells	Up	DAPK1	Prognosis	[116,117]
hsa-miR-200a	Promotes EMT of EC cells by negatively regulating FOXA2 expression	ET	Up	FOXA2	Diagnosis	[118]
hsa-miR-15a-5p	Inhibits the growth of EC cells via attenuating WNT3A expression in the Wnt/β-catenin signaling pathway.	- ET - Cell lines: HEC-251, AN3CA, RL95-2, HEC-1-A, ISK, Ishikawa, and JEC	Down	WNT3A	Diagnosis	[47]
hsa-miR-449a	Might module the EC progression via negative regulation of transcription from RNA polymerase II promoter	ET	Down	LEF1	Prognosis	[48]
hsa-miR-145-5p	Might act as a tumor suppressor and regulate cell cycle associated processes to inhibit the development of EC	ET	Down	SOX11	Prognosis	[48]
hsa-miR-505	Functions as a tumor suppressor by targeting TGF-α	- ET - EC cell lines: HEC-1B and Ishikawa	Down	TGF-α	Diagnosis	[49]
hsa-miR-589-5p	Inhibits cell proliferation, migration, and invasion by targeting TRIP6.	- ET - EC cell lines: HEC-1B and AN3CA	Down	TRIP6	Diagnosis	[50]
hsa-miR-20a-5p	- Inhibits EMT and invasion of EC cells by targeting STAT3 - Inhibits EC progression by targeting Jak1.	- EC cell lines: ECC-1, KLE, HHUA, RL95-2 and Ishikawa - HEK293 cells - Human uterine epithelial cell line HES - ET	Down	STAT3 Jak1	Diagnosis	[51,52]
hsa-miR-23b	It may act as a tumor suppressor miR: suppressed the proliferation of Ishikawa cells.	- ET - Ishikawa EC cells	Down	CCNG1	Prognosis	[46,119]

EC: endometrial cancer, ET: endometrial tissue, MAPK1: mitogen-activated protein kinase, Bcl-2: B cell lymphoma-2, FAM98A: family with sequence similarity 98 members A, NIFK-AS1: NIFK antisense RNA 1, SNAI2: snail family transcriptional repressor 2, FGFR1: acidic fibroblast growth factor receptor FGF2: basic fibroblast growth factor, PI3K: phosphatidylinositol 3-kinase, AKT: protein kinase B, ERK: extracellular regulated MAP kinase, VEGFA: vascular endothelial growth factor A, HIF1A: hypoxia inducible factor 1 A, PTEN: phosphatase and tensin homolog, STAT3: signal transducer and activator of transcription 3, FOXC1: forkhead box C1, KLF4: kruppel-like factor 4, VAV3: vav guanine nucleotide exchange factor 3, FOXO1: forkhead Box O1, EMT: epithelial mesenchymal transition, HOXB3: homeobox box 3, SOX4: SRY-related high-mobility group box 4, GPER or GPR30: G protein-coupled estrogen receptor, UBE3C: ubiquitin protein ligase E3C, BECN1: Beclin 1, AURKB: aurora kinase B, LMOD1: leiomodin 1, ERα: estrogen receptor alpha, MDM2: MDM2 proto-oncogene, HIF1-α: hypoxia inducible factor-1 alpha, RAMP1: receptor activity modifying protein 1, ING5: inhibitor of growth family member 5, ENPP2: ectonucleotide pyrophosphatase/phosphodiesterase 2, SNCAIP: synuclein, alpha interacting protein, CDC25B: cell division cycle 25B, mTOR: mechanistic target of rapamycin kinase, 4E-BP1: eukaryotic translation initiation factor 4E binding protein 1, GPRASP2: G protein-coupled receptor associated sorting protein 2, MTA1: metastasis-associated protein 1, KLF2: kruppel-like factor 2, Rab25: ras-related protein Rab-25, TGFB1I1: transforming growth factor beta 1 induced transcript 1, BCL2L11: BCL2 similar to 11, DPP6: dipeptidyl-peptidase 6, C2orf32: chromosome 2 open reading frame 32, NFIX: nuclear factor I/X, ZO-1: tight junction protein 1, TIMP-3: tissue inhibitor of metalloproteinase 3, SEC: serous endometrial cancer, KLF9: kruppel-like factor 9, BRD7: bromodomain containing 7, MMP-9: matrix metallopeptidase 9, DAPK1: death-associated protein kinase 1, FOXA2: forkhead box A2, WNT3A: wnt family member 3A, LEF1: lymphoid enhancer binding factor 1, SOX11: SRY-box transcription factor 11, TGF-α: transforming growth factor-α, TRIP6: thyroid receptor interacting protein 6, Jak1: janus kinase 1, CCNG1: cyclin G1. * The specific role in EC was not identified in any of the studies consulted. The references from 82 to 114 are reported exclusively in Table 3.
